# Baseline diet quality of predominantly minority children and adolescents from households characterized by low socioeconomic status in the Childhood Obesity Prevention and Treatment Research (COPTR) Consortium

**DOI:** 10.1186/s40795-019-0302-y

**Published:** 2019-09-09

**Authors:** Kimberly P. Truesdale, Donna M. Matheson, Meghan M. JaKa, Sarah McAleer, Evan C. Sommer, Charlotte A. Pratt

**Affiliations:** 1 Department of Nutrition, University of North Carolina at Chapel Hill, Chapel Hill, NC 27599 USA; 2 Department of Pediatrics, School of Medicine, Stanford University, Palo Alto, CA USA; 3 Health Partners Institute for Education and Research, Minneapolis, MN USA; 4 The Center for Child Health and Policy, Rainbow Babies & Children’s Hospital, Case Western Reserve University, Cleveland, OH USA; 5 Department of Pediatrics, Vanderbilt University Medical Center, Nashville, TN USA; 6 Division of Cardiovascular Sciences, National Heart, Lung, and Blood Institute (NHLBI), National Institute of Health, Bethesda, MD USA

**Keywords:** Diet quality, Children, Obesity, Body mass index

## Abstract

**Background:**

The Healthy Eating Index (HEI-2010) is a measure of diet quality that examines conformance with the Dietary Guidelines for Americans. The objectives of this study were to estimate baseline diet quality of predominantly low-income minority children using the HEI-2010 and to identify the most important HEI components to target for dietary intervention.

**Methods:**

Two or three baseline 24 h dietary recalls were collected in-person or over telephone between May 2012 and June 2014 from 1,745 children and adolescents from four randomized clinical trials in the Childhood Obesity Prevention and Treatment Research (COPTR) Consortium. Nine adequacy and three moderation food components were calculated and averaged to determine overall HEI scores. The overall HEI-2010 scores were categorized as ≥81, 51–80, or ≤ 50 based on the HEI-2005 classification. For each study, mean overall and component HEI scores were estimated using linear regression models.

**Results:**

Mean (95% CI) overall HEI scores ranged from 47.9 (46.8, 49.0) to 64.5 (63.6, 65.4). Only 0.3 to 8.1% of children and adolescents had HEI-2010 score ≥ 81. The average component score for green and beans was less than 30% of maximum score for all trials. In contrast, the average component score for protein, dairy (except for IMPACT), and empty calories (except forIMPACT) was more than 80% of maximum score.

**Conclusions:**

Based on HEI-2010 scores, few children and adolescents consumed high quality diets. Dietary interventions for children and adolescents should focus on improving intakes of green vegetables and beans.

**Clinical trial registry numbers:**

GROW study (clinical trial # NCT01316653); NET-Works study (clinical trial #NCT01606891); Stanford Goals (clinical trial #NCT01642836); IMPACT (clinical trial # NCT01514279).

## Background

Diet quality has been well recognized as a multifaceted construct [1–3]. A number of dietary quality indexes have been developed and used in different settings including the Healthy Eating Index (HEI) [4], Alternative Healthy Eating Index, Diet Quality Index [5], Recommended Food Score [6, 7] and Alternative Mediterranean Diet Index. The HEI is designed to assess conformance with the recommendations of the Dietary Guidelines for Americans [4, 8]. It is updated every 5 years based on refinements to the U.S. Dietary Guidelines. Guenther et al. used exemplary menus and 24-h dietary data from the 2003–2004 *National Health and Nutrition Examination Survey (NHANES) to examine the validity and reliability of the HEI-2010* [9]. Analysis of the four exemplary menus produced very high HEI 2010 scores (87.8–100) thus validating the ability to identify high diet quality [9]. The HEI 2010 scores from the NHANES data had a wide range which enable researchers to detect meaningful changes in HEI 2010 scores and compare diet quality between groups [9]. In addition, the HEI 2010 overall and components scores were independent of energy intake [9].

The HEI 2010 [10] has 12 components with the total score ranging from 0 to 100 and a score of ≥81 indicating good diet quality [11]. The mean HEI score for children from the 2011–2012 NHANES data were substantially lower than the cutoff for a good quality diet (mean = 50.9, (95% CI: 50.5, 51.8)) [12]. The three component scores that were particularly low were total vegetables, greens and beans and whole grains.

This paper reports the baseline dietary quality of children and adolescents in the Childhood Obesity Prevention and Treatment Research (COPTR) Consortium. The objectives of this study were to: 1) assess the baseline diet quality of the children using the HEI 2010 and 2) determine if the diet quality of the COPTR children differ by sex, age, race/ethnicity, weight status, parent’s marital status, employment status or SNAP participation. It was hypothesized that most COPTR children would have HEI scores below 80, indicating that they do not consume diets that meet the U.S. Department of Agriculture (USDA) dietary recommendations [13]. Second, it was hypothesized that low total HEI scores would be due to low intakes of fruits and vegetables. Additionally, it was hypothesized that HEI scores would differ by age, race/ethnicity and weight status but not by sex.

## Methods

### Study design and participants

The COPTR Consortium is comprised of four independent randomized controlled trials (RCT) of childhood obesity prevention or treatment. Each RCT implemented a 3-year intervention that was unique and different, but used common and standardized data collection procedures. Additional details of the COPTR Consortium and each intervention study have previously been published [14–18]. The University of North Carolina at Chapel Hill serves as the Research Coordinating Center and receives all common data for the sites. The two childhood obesity prevention trials are located in Minneapolis, MN (University of Minnesota, Now Everyone Together for Healthy and Amazing Kids (NET-Works) Study) and Nashville, TN (Vanderbilt University, Growing Right Onto Wellness (GROW) Study), and the two childhood obesity treatment trials are located in Cleveland, OH (Case Western Reserve University, Ideas Moving Parents and Adolescents to Change Together (IMPACT Study) and Bay Area, CA (Stanford University, Stanford GOALS Study). This study was approved by the Institutional Review Boards on research involving human subjects at University of North Carolina at Chapel Hill, University of Minnesota, Vanderbilt University, Stanford University and Case Western Reserve University.

All four studies recruited predominantly minority populations from households with low socioeconomic status. The sample size, recruitment age range and weight status varied for the four studies. The NET-Works Study recruited 534 2–4 year old children at or above 50th BMI percentile. The GROW Study recruited 610 3–5 year old children between the 50th and 94.9th BMI percentile. The IMPACT Study recruited 360 rising 6th graders at or above the 85th BMI percentile. The Stanford GOALS Study recruited 241 7–11 year old children at or above the 85th BMI percentile. If the household had more than one child that met the eligibility criteria (e.g. two children between 7 and 11 years of age above the 85th BMI percentile) then only one child was randomly selected to be in the study. These analyses are conducted with baseline data from each site. Baseline data were collected between May 2012 and June 2014. For each study, parental consent was obtained for minor child to participate in the study. The two studies with older children also obtained written assent from children.

#### Dietary assessments

Dietary intakes at all sites were measured using 24-h recalls that were collected on two weekdays and one weekend day using the Nutrition Data System for Research (NDSR) software [19–21]. NDSR versions 2011, 2012 and 2013 were used. Dietary recalls were conducted by trained and certified NDSR interviewers. Bilingual (English and Spanish) interviewers conducted dietary recalls in Spanish when requested. The first dietary recall was conducted in-person (except for GROW) and the second (except for NET-Works) and third dietary recalls were conducted over the telephone. In older children (IMPACT and GOALS), the child self-reported their dietary intake with parental assistance, when needed (e.g. provided details on how a food item was prepared). In the preschool-aged samples (NET-Works and GROW), the parent/guardian served as a proxy for the child to report the child’s previous day intake. Food amounts booklets were used by the respondent to assist in identifying portion sizes. For children in childcare, food records were given to the childcare provider and the completed form was used by the parent to report foods the child consumed while in childcare. School menus were also used when needed. The percentage of participants with three dietary recalls was 97.6% in NET-Works, 64.4% in GROW, 96.1% in IMPACT and 100% in GOALS. Average intakes of energy, macro- and micronutrients and food groups were calculated based on the average of each participant’s diet recalls (2 or 3 days).

Dietary quality was measured using the 2010 Healthy Eating Index [10]. The HEI-2010 was used since it aligns with the 2010–2015 Dietary Guidelines for Americans and the data were collected during this time period. The HEI is comprised of nine adequacy and three moderation food components with a predefined maximum score (5, 10, 20) per component. The maximum scores assigned for each component are based on the USDA recommended daily intake per 1,000 kcal (exception: fatty acid component and empty calories component are not standardized to 1,000 kcal). The overall HEI score is the summation of the 12 component scores and ranges from 0 to 100 points with higher scores indicating better dietary quality. Based on the USDA HEI-2005 grading scale, the child’s diet quality was categorized into three groups, 0–50, 51–80 and 81–100) [11]. The Nutrition Coordinating Center (NCC) guidelines and SAS macro for NDSR data were used to calculate the HEI-2010 scores with one exception [22]. Empty calories are the calories from solid fats, alcohol and added sugars. Prior to NDSR version 2014, calories from solid fats were not calculated in the NDSR software, therefore, the individual’s food intakes were used to calculate grams and calories from solid fat. Following the NCC guidelines and SAS macro [22], a component score was calculated for each recall then averaged (2 or 3 recalls) to determine the average HEI component scores.

#### Covariates

Weights and heights of index parents and children were measured with the participant in light clothing, without shoes, using a standardized protocol across all sites. Weight was measured to the nearest 0.1 kg using research precision grade, calibrated, digital scales and height was measured to the nearest 0.1 cm using a free-standing or wall mounted stadiometer. BMI was calculated as weight in kilograms divided by height in meters squared and age and sex specific BMI percentiles were calculated using the CDC macro [23] and used to categorized children as high normal weight (50th – 84.9th BMI percentile), overweight (85th – 94.9th BMI percentile) or obese (≥95th BMI percentile) [24, 25]. The index parent/guardian was categorized as either underweight (< 18.5 kg/m^2^), normal weight (≥18.5- < 25.0 kg/m^2^), overweight (≥25.0 - < 30.0 kg/m^2^) or obese (≥30.0 kg/m^2^) [26].

Race/ethnicity, age (date of birth) and sex of index child, marital status (married/living as married, single), employment status (full time, part time, not working for pay) of the index parent/guardian, highest level of household education (<high school, high school or equivalent, at least some college) and participation in supplemental nutrition assistance program (SNAP) were self-reported. Children were classified into five mutually exclusive race/ethnicity groups (Non-Hispanic White, Non-Hispanic Black, Hispanic, multi-racial or other) based on their self-reported race and ethnicity. All questionnaires were administered in English or Spanish according to participant’s preference.

#### Exclusions

Participants with less than two reliable dietary recalls in a 45-day window (GROW: *n* = 1; IMPACT: n = 1) or BMI percentile was outside the pre-defined recruitment inclusion criteria (GROW: *n* = 7) were excluded from the analysis. Reliability of the dietary recall was determined by the interviewer based on the interviewee’s ability to recall dietary intake from previous day. The analytical sample size was 534 for NET-Works, 602 for GROW, 241 for GOALS and 359 for IMPACT.

#### Statistical analysis

The samples were independently recruited so all analyses were conducted separately. For each participant, the mean overall and 12 component HEI scores were calculated for each recall then averaged. In order to identify potential dietary intervention targets, the percent of the maximum component score was calculated by dividing the average HEI component score by the maximum component score. Linear regression models were used to determine if the overall HEI score differed by six key demographic variables -sex, age, BMI percentile, marital status, employment status and SNAP participation. Because there was more variability in race/ethnicity in the NET-Works Study, this variable (NH White, NH Black, Hispanic, Multi/Other) was also included in the regression models for this site. The fully adjusted models included all of the key demographic variables. The least squares mean HEI scores for the levels of the demographic variables were compared. All analyses were conducted in SAS version 9.4 (SAS Institute) [27].

## Results

The demographic baseline characteristics of the four studies are shown in Table 1. All four studies recruited and randomized predominantly minority (87.4–100%) children. The children in the GROW and GOALS Studies were mostly Hispanic; whereas, the IMPACT Study was mostly African American children and the NET-Works study had a mix of non-Hispanic Whites, Blacks and Hispanics. The mean BMI percentile in each study reflects the eligibility criteria of each study. In general, the index parent tended to be in their 30s (~ 32 years of age for prevention studies and 37–38 years of age for treatment studies), have BMI ≥ 25.0 kg/m^2^, married or living as married (except for IMPACT Study), with HS education or less (except for IMPACT Study) and not working for pay.

**Table 1:** Baseline demographic characteristics of the participants by Childhood Obesity Prevention and Treatment Research (COPTR) study, May 2012–June 2014

	Prevention Sites				Treatment Sites			
	NET-Works		GROW		IMPACT		Stanford GOALS	
	Minneapolis, Minnesota (*n* = 534)		Nashville, Tennessee (*n* = 602)		Cleveland, Ohio (*n* = 359)		East Palo Alto, California (*n* = 241)	
	Mean or % SD		Mean or % SD		Mean or % SD		Mean or % SD	
Index child								
Age (years)	3.4	0.7	4.3	0.9	11.6	0.6	9.5	1.4
Race/Ethnicity								
Non-Hispanic white	12.6		1.0		3.9		0.0	
Non-Hispanic black	18.4		6.0		76.9		1.7	
Hispanic	58.4		90.9		16.2		97.9	
Multi-racial	8.4		1.0		2.2		0.0	
Other^a^	2.2		1.2		0.8		0.4	
Sex (% female)	50.9		52.0		57.9		55.6	
BMI %tile	81.7	14.3	77.1	12.7	95.7	3.7	96.5	3.2
BMI status^b^								
Upper normal weight	51.7		64.9		0.0		0.0	
Overweight	25.7		33.9		32.8		24.1	
Obese	22.7		1.2		67.2		75.9	
Total calories	1046.4	327.4	1194.3	384.6	1437.5	460.1	1194.0	405.2
Index parent								
Age (years)	31.9	6.4	32.1	6.0	37.6	8.1	36.7	6.9
Sex (% female)	91.7		98.3		95.2		94.7	
BMI status^c^								
Underweight	1.0		0.3		0.9		0.4	
Normal weight	23.2		19.6		9.1		12.8	
Overweight	30.6		39.0		17.2		31.3	
Obese	45.2		41.0		72.8		55.5	
Education								
< High School	39.9		61.5		20.6		71.4	
HS grad/equivalent	20.2		20.6		27.3		13.3	
≥ Some college/trade school	39.9		17.9		52.1		15.4	
Marital status (% single)	31.3		17.0		66.7		14.1	
Employment status								
Full time	29.8		18.0		37.9		32.4	
Part time	27.7		19.6		17.8		24.1	
Not working for pay	42.5		62.4		44.3		43.6	
SNAP % yes)	43.0		75.3		70.5		40.7	

^a^Other race/ethnicities are Asian, American Indian, Alaskan Native, Native Hawaiian or Pacific Islander

^b^Index child BMI status categories are upper normal weight (≥50th - <85th BMI percentile), overweight (≥85th - <95th BMI percentile) and obese (≥95th BMI percentile) [24, 25]

^c^Index parent BMI status categories are underweight (> 18.5 kg/m^2^), normal weight (≥18.5 - < 25.0 kg/m^2^), overweight (≥25.0 - < 30.0 kg/m^2^) and obese (≥30.0 kg/m^2^) [26]

**Fig. 1:**
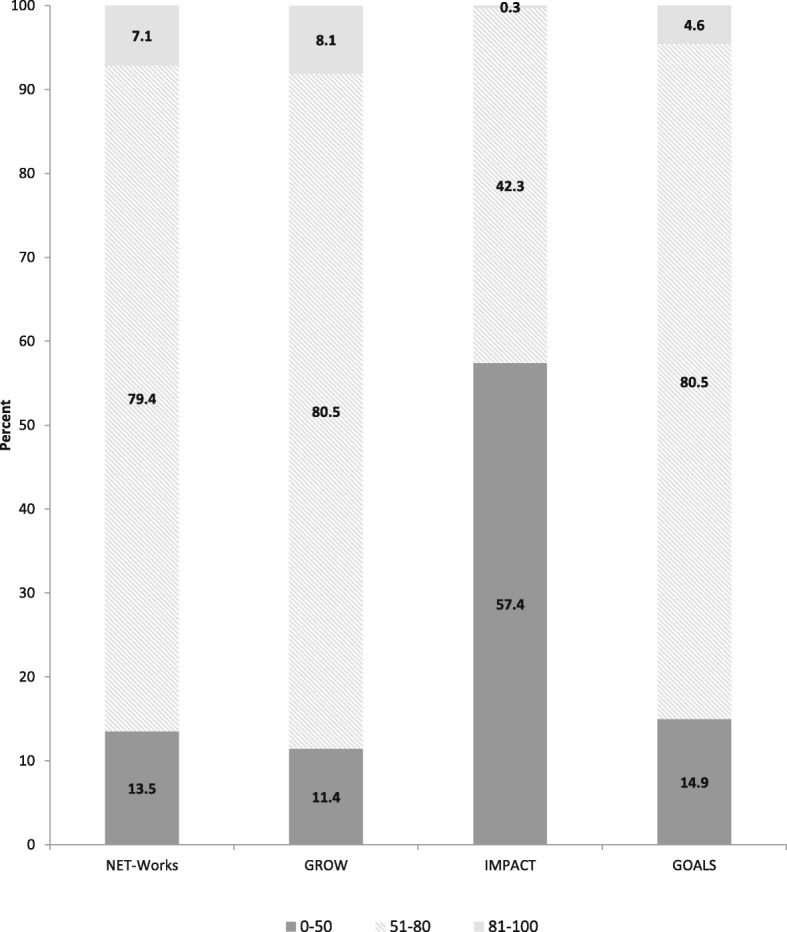
Distribution of the HEI-2010 scores by by Childhood Obesity Prevention and Treatment Research (COPTR) study, May 2012–June 2014. HEI-2010 scores were divided into three categories: 0–50, 51–80 and 81–100

Table 2 shows the mean (95% CI) overall and component HEI scores. The mean HEI score was 63.7 (95% CI: 62.8, 64.7), 64.5 (95% CI: 63.6, 65.4), 47.9 (95% CI: 46.8, 49.0) and 61.7 (95% CI: 60.3, 63.2), for the NET-Works, GROW, IMPACT and GOALS studies, respectively. Approximately 7–8% of the children in the preschool-aged studies (NET-Works and GROW) and 4.6% of adolescent-age children in the GOALS study had HEI score ≥ 81 and about 80% had a HEI score between 51 and 80 (Fig. 1). In contrast in the IMPACT study, only 0.3% (*n* = 1 adolescent-aged child) had a HEI score ≥ 81. The majority of the adolescents from the IMPACT study (57.4%) had HEI ≤ 50.

**Table 2:** Mean (95% CI) overall and component healthy eating index (HEI) scores and percentage of subjects with the maximum and minimum number of points for each component by Childhood Obesity Prevention and Treatment Research (COPTR) Study, May 2012–June 2014

	NET-Works	% of max score	GROW	% of max score	IMPACT	% of max score	GOALS	% of max score
	Mean (95% CI)		Mean (95% CI)		Mean (95% CI)		Mean (95% CI)	
Total Score (100)	63.7 (62.8, 64.7)	64	64.5 (63.6, 65.4)	64	47.9 (46.8, 49.0)	48	61.7 (60.3, 63.2)	62
Total vegetables (5)	2.0 (1.9, 2.1)	40	2.1 (2.0, 2.2)	42	1.9 (1.8, 2.0)	38	2.3 (2.2, 2.5)	40
Greens and beans (5)	1.2 (1.0, 1.3)	24	1.2 (1.1, 1.4)	24	1.2 (1.0, 1.4)	24	1.5 (1.3, 1.8)	30
Total fruit (5)	3.9 (3.7, 4.0)	78	4.0 (3.9, 4.1)	80	1.9 (1.8, 2.1)	38	3.1 (2.9, 3.4)	62
Whole fruit (5)	3.9 (3.7, 4.0)	78	3.9 (3.8, 4.0)	78	1.6 (1.4, 1.8)	32	3.4 (3.2, 3.6)	68
Whole grains (10)	5.4 (5.1, 5.7)	54	5.7 (5.4, 6.0)	57	3.0 (2.7, 3.3)	30	6.2 (5.7, 6.6)	62
Dairy (10)	8.6 (8.4, 8.8)	86	8.8 (8.6, 9.0)	88	6.0 (5.7, 6.3)	60	8.6 (8.3, 8.8)	86
Total protein foods (5)	4.1 (4.1, 4.2)	82	4.2 (4.1, 4.2)	84	4.6 (4.5, 4.7)	92	4.3 (4.2, 4.5)	86
Seafood and plant protein (5)	2.0 (1.9, 2.2)	40	2.1 (1.9, 2.2)	42	1.2 (1.0, 1.4)	24	1.7 (1.5, 2.0)	34
Fatty acid ratio (5)	3.2 (2.9, 3.5)	32	2.8 (2.6, 3.1)	32	4.5 (4.2, 4.8)	32	3.2 (2.8, 3.6)	32
Sodium (10)	5.9 (5.6, 6.1)	59	6.0 (5.8, 6.3)	60	3.2 (2.9, 3.5)	32	4.5 (4.1, 4.9)	45
Refined grains (10)	7.3 (7.1, 7.6)	73	7.8 (7.5, 8.0)	78	4.8 (4.5, 5.2)	48	5.5 (5.1, 5.9)	55
Empty calories (20)	16.3 (15.9, 16.6)	82	15.9 (15.6, 16.2)	80	14.0 (13.5, 14.5)	70	17.3 (16.9, 17.7)	87

The average component score for the greens and beans component was 24% of the maximum score for the NET-Works, GROW, and IMPACT studies and 30% of the maximum score for the GOALS study. In addition, the average component score was less than half of the maximum score in all studies for three component scores – total vegetables, seafood and plant protein, and fatty acid ratio. There were an additional 5 HEI components that scored less than 50% of the maximum score for the IMPACT study (total fruit, whole fruit, whole grains, sodium, and refined grains). In contrast, the average component score for total protein (all studies), dairy (all except IMPACT), empty calories (all except IMPACT) and total fruit (GROW only) was at least 80% of the maximum score.

**Fig. 2:**
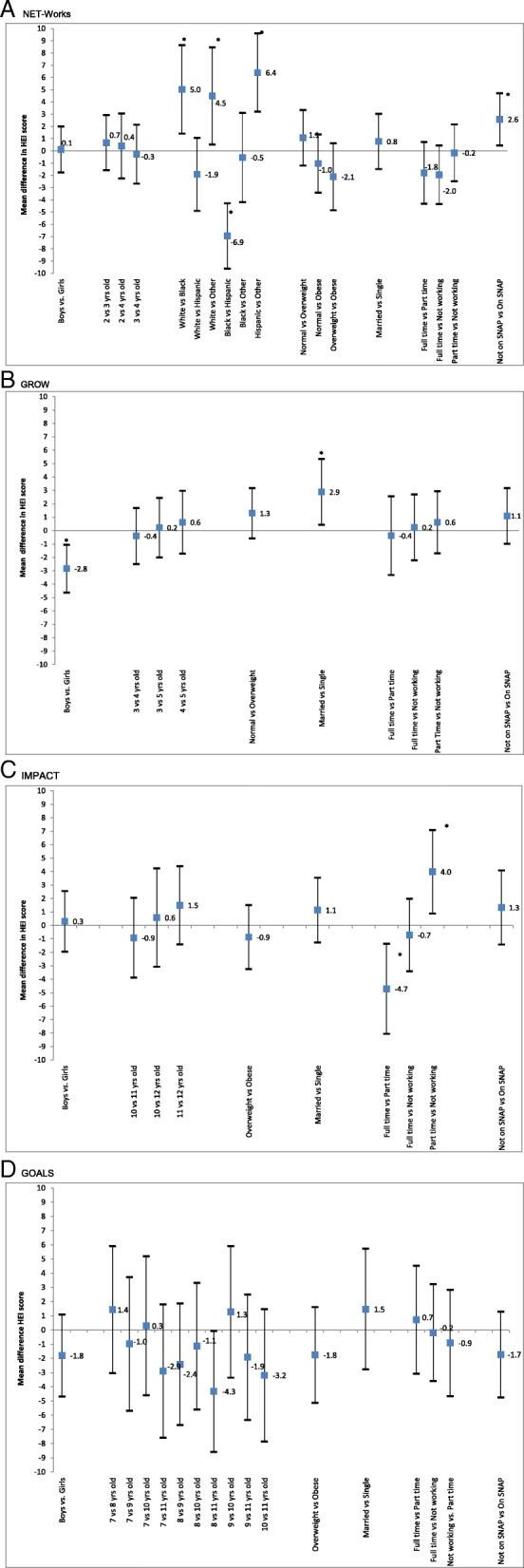
Adjusted differences in the HEI-2010 total score by key sociodemographic characteristics by Childhood Obesity Prevention and Treatment Research (COPTR) study, May 2012–June 2014. Panel A is NET-Works, Panel B is GROW, Panel C is IMPACT and Panel D is GOALS. * Significant difference (*p* < 0.05)

Figure 2 shows the adjusted differences in mean overall HEI scores by key sociodemographic characteristics. The mean HEI scores did not vary significantly by the child’s age for any of the studies. In the NET-Works study (Fig. 2a), with respect to the mean HEI score, White children scored a mean of 5.0 (95% CI: 1.4, 8.6) points higher than non-Hispanic black children and 4.5 (95% CI: 0.5, 8.5) points higher than other children. Black children mean HEI score was 6.9 (95% CI: − 9.6, − 4.3) points lower than Hispanic children and Hispanic children mean HEI score was 6.4 (95% CI: 3.2, 9.6) points higher than other children. Children who were not receiving benefits from SNAP HEI scores were 2.6 (95% CI: 0.5, 4.7) points higher than children participating in SNAP. Similar patterns, although not significant, were observed for SNAP participation in the GROW (Fig. 2b) and IMPACT (Fig. 2c) studies. In the GROW study of predominantly pre-school aged Hispanic children (Fig. 2b), the mean HEI score differed by sex (boys lower than girls; mean difference: -2.8, 95% CI: − 4.6, − 1.1). Also in the GROW study, children living in married/living as married households mean HEI score was 2.9 (95% CI: 0.4, 5.3) points higher than children living in single status households. Similar patterns (but not significant) for sex and marital status were observed in the predominantly older Hispanic children in the GOALS study (Fig. 2d). In the IMPACT study (Fig. 2c), children whose index parent worked full-time mean HEI score was 4.7 points lower than children whose index parent worked part-time (95% CI: − 8.1, − 1.4) and children whose index parent worked part-time mean HEI score was 4.0 (95% CI: 0.9, 7.1) points higher than children whose index parent did not work for pay.

## Discussion

The primary objective of this study was to examine the baseline diet quality of the children in the COPTR Consortium. Less than 10% of the children consumed a diet with total HEI scores ≥81. The mean HEI scores ranged from 47.9 (IMPACT study) to 64.5 (GROW study). Approximately 80% of the children from the NET-Works, GROW and GOALS studies HEI scores were between 51 and 80. These studies had 58.4, 90.9 and 97.9% Hispanic population, respectively. In contrast, 99.7 children from the IMPACT study (76.9% African American) scored less than 81 points with the majority scoring less than 51 points.

### Vegetable consumption

Total vegetable consumption was low in children and adolescents from all four studies with less than half the maximum points scored. This is consistent with the literature which estimates that 93% of US children and adolescents do not meet the recommended daily amounts for vegetables [28] and the total vegetable component percent of the maximum score ranged was 40–42% in children 2–13 years of age [29]. Furthermore, previous research has found that about 30% of vegetable intake is from white potatoes [28, 30]. In the current study, less than 30% of the maximum score was achieved for the greens and beans component. This is slightly higher that findings from NHANES for similar aged children (18–24%) [29]. In general, both component scores (greens and beans and total vegetables) and overall HEI score could potentially improve with greater consumption of greens and beans.

### Protein sources

In these four predominantly minority children from households characterized by low socioeconomic status, the average component score for protein was more than 80% of the maximum score. However, the average component score for seafood or plant based protein was only 24–42% of the maximum score. Previous research has linked higher consumption of lean meat and seafood with higher socioeconomic status [31]. Lower SES groups consumed more fried or canned seafood. Increasing overall red meat, pork and poultry intake will not impact children’s overall diet quality as much as increasing consumption of seafood and plant based proteins.

### Fruits and whole fruits

In our two preschool studies, the average component score was 78–80% of the maximum score for the total fruit and whole fruit components and 86–88% of the maximum score for the dairy component. Previous research has shown that both fruit [32, 33] and diary consumption are higher in younger children and declines as the children get older. Different patterns were observed in the adolescent children in the treatment studies. In the GOALS study, where children were 7 to 11 years of age at baseline, the average component score was 62–68% of the maximum score for the total fruit and whole fruit components and 86% of the maximum score for the dairy component. In contrast, the children in the IMPACT study (10–13 years old) average component score for the total fruit, whole fruit and dairy components was 38, 32 and 60% of maximum score, respectively.

### Racial/ethnic differences

The NET-Works study is the only study with adequate diversity in multiple race/ethnic groups to examine differences. We found significantly lower mean HEI scores in African American children compared to Hispanic children. The findings from the NET-Works study is in concordance with the lower HEI scores in found in the predominantly African American adolescents in the IMPACT study compared to relatively higher HEI scores in the predominantly Hispanic children in the GROW and GOALS studies. These findings of lower diet quality scores in African American children than in Hispanic children are consistent with the literature [34].

The diet intake of African American children has been shown to be poor and does not meet recommendations for whole fruits, total vegetables, whole grains, and dairy [35, 36]. Furthermore, it is important to remember, based on data from the NHANES 2011–2012, that the dietary intake of most American children is not in the high quality range (mean overall HEI score of 50.9, 95% CI: 50.5, 51.8), yet African American children have an even lower mean score (48.4, 95% CI: 46.9–49.9) [12]. This highlights promising target areas for intervention efforts seeking to improve the overall diet quality in predominantly African American children who are overweight or obese and from households characterized by low socioeconomic status.

In the current study, the relationships between social demographic characteristics and HEI across were not consistent across all four studies. These inconsistent findings may be due to the differences in the race/ethnicity, age, and/or BMI distribution in the four studies. Previous research using the HEI-2005 has shown that preschool-aged children had a higher diet quality than adolescent-aged children [34]. Gu et al. also found similar results in the NHANES 2011–2012 dataset where the mean HEI 2010 scores were 55.3 (95% CI: 53.6. 57.0), 51.2 (95% CI: 49.5, 52.9), and 48.4 (95% CI: 47.0, 49.8) for 2–5, 6–11 and 12–18 year olds, respectively [12]. Gu et al. [12] and Hiza et al. [34] also found that girls had a slightly higher diet quality than boys, though this difference was not statistically significant. Hispanic children had a higher diet quality than non-Hispanic black children, but no other racial or ethnic differences were seen [34]. In addition, Hiza et al. found a non-linear relationship between family income and diet quality, with children in the lowest income group and those in the highest income group having higher diet quality than those in the middle groups [34]. These differences seemed to be driven primarily by differences in fruit intake, though milk intake was a key factor in the diet quality differences by age. While the current study did not identify any demographic characteristic as a target for the total HEI score, there may be associations for specific components. It is important to determine the HEI components where the average component score is a low percentage of the maximum score to identify potential targets for dietary intervention.

### Strengths and limitations

One of the strengths of this study is that diet quality was assessed in four studies of predominantly minority children from households characterized by low socioeconomic status using 24-h dietary recalls. Data were analyzed separately for each study to determine if any patterns were consistent across the four populations. Another strength of this research is that HEI components that could be targets for future nutrition interventions in low-income minority populations were identified. Across all studies, the overall HEI scores would increase in the vast majority of children by increasing consumption of green vegetables, beans, seafood and plant based protein.

One limitation of this study is that three of the four COPTR studies did not recruit a variety of race/ethnic groups. Therefore, race/ethnic comparisons could not be made in all studies. The NET-Works study did have multiple race/ethnic groups and significant differences were found between Whites, African Americans and Hispanics. Hispanics are a heterogeneous group and in the current study we were unable to separate Hispanics by country of origin. In the NET-Works, GROW and GOALS studies, the majority of Hispanics were Mexican Americans (74, 71 and 85%) and in the IMPACT study the majority (56%) were from Puerto Rico. Another limitation of this study is that the analysis did not correct for episodically consumed foods or examine seasonality. Baseline dietary data were collected in every calendar month (except do data collected in January or February for IMPACT). The HEI scores were based on 2 or 3 24-h dietary recalls and may not represent usual intake for all food groups.

## Conclusion

In summary, a small percentage of predominantly minority children from households characterized by low socioeconomic status consumed a good quality diet (HEI ≥ 81) at baseline. Future dietary intervention efforts should target seafood or plant based protein, legumes, greens that are limited in the diets of children and adolescents in order to improve their overall diet quality.

## Data Availability

The datasets generated and/or analyzed during the current study are not publicly available. The COPTR data will be made publicly available in July 2020 (as required by NIH) via Biolincc.

## Abbreviations

BMI: Body Mass Index
CDC: Centers for Disease Control and Prevention
COPTR: Childhood Obesity Prevention and Treatment Research
GROW: Vanderbilt University, Growing Right Onto Wellness
HEI: Healthy Eating Index
IMPACT: Ideas Moving Parents and Adolescents to Change Together
NCC: Nutrition Coordinating Center
NDSR: Nutrition Data System for Research
NET-Works: Now Everyone Together for Healthy and Amazing Kids
NHANES: National Health and Nutrition Examination Survey
RCT: Randomized controlled trials
SNAP: Supplemental nutrition assistance program
USDA: U.S. Department of Agriculture
